# Comparison of an oral mixed meal plus arginine and intravenous glucose, GLP-1 plus arginine to unmask residual islet function in longstanding type 1 diabetes

**DOI:** 10.1152/ajpendo.00030.2024

**Published:** 2024-03-06

**Authors:** Bas S. Uitbeijerse, Michiel F. Nijhoff, Eelco J. P. de Koning

**Affiliations:** Department of Internal Medicine, Leiden University Medical Center, Leiden, The Netherlands

**Keywords:** alpha cell, beta cell function, glucose clamp, human, type 1 diabetes

## Abstract

Residual beta cells are present in most patients with longstanding type 1 diabetes but it is unknown whether these beta cells react normally to different stimuli. Moreover a defect in proinsulin conversion and abnormal alpha cell response are also part of the islet dysfunction. A three-phase [euglycemia, hyperglycemia, and hyperglycemia + glucagon-like peptide 1 (GLP-1)] clamp was performed in patients with longstanding type 1 diabetes. Intravenous arginine boluses were administered at the end of each phase. On another day, a mixed meal stimulation test with a subsequent intravenous arginine bolus was performed. C-peptide was detectable in a subgroup of subjects at baseline (2/15) or only after stimulation (3/15). When detectable, C-peptide increased 2.9-fold [95% CI: 1.2–7.1] during the hyperglycemia phase and 14.1-fold [95% CI: 3.1–65.2] during the hyperglycemia + GLP-1 phase, and 22.3-fold [95% CI: 5.6–89.1] during hyperglycemia + GLP-1 + arginine phase when compared with baseline. The same subset of patients with a C-peptide response were identified during the mixed meal stimulation test as during the clamp. There was an inhibition of glucagon secretion (0.72-fold, [95% CI: 0.63–0.84]) during the glucose clamp irrespective of the presence of detectable beta cell function. Proinsulin was only present in a subset of subjects with detectable C-peptide (3/15) and proinsulin mimicked the C-peptide response to the different stimuli when detectable. Residual beta cells in longstanding type 1 diabetes respond adequately to different stimuli and could be of clinical benefit.

**NEW & NOTEWORTHY** If beta cell function is detectable, the beta cells react relatively normal to the different stimuli except for the first phase response to intravenous glucose. An oral mixed meal followed by an intravenous arginine bolus can identify residual beta cell function/mass as well as the more commonly used glucose potentiated arginine-induced insulin secretion during a hyperglycemic clamp.

## Graphical abstract

**Figure:**
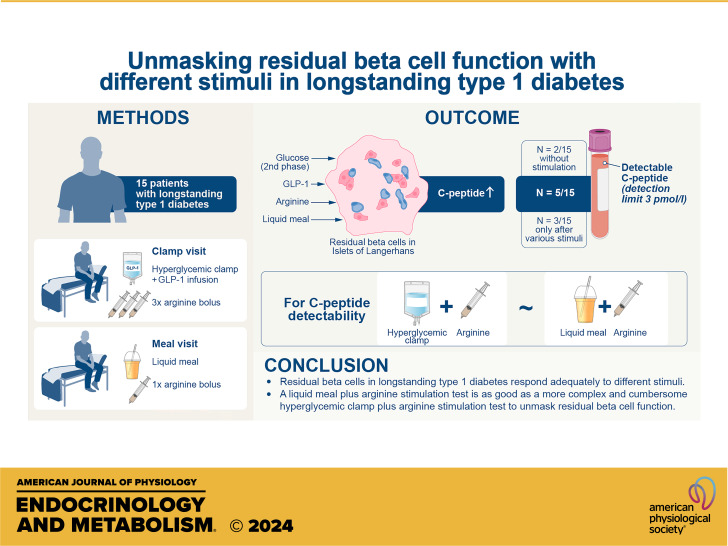

## INTRODUCTION

Beta cells are identified in 88% of pancreases in individuals with type 1 diabetes who have been diagnosed 4–67 years earlier (1). Circulating C-peptide as a measure of beta cell function are detected in up to 73% of patients with longstanding type 1 diabetes, depending on the population investigated and the sensitivity of the C-peptide assay (2–5). This persisting beta cell function is associated with better glycemic control, a lower risk of retinopathy and albuminuria, and a lower risk of hypoglycemia (6, 7).

Insulin secretion is dynamically regulated with a major role for carbohydrates and meal-related incretin hormones. Therefore, the standardized mixed meal stimulation test [also called mixed meal tolerance tests (MMTT)] is often used, showing a beta cell secretory response in many patients with longstanding type 1 diabetes (3–5). However the results of stimulated C-peptide during this test are affected by concurrent glucose concentration and insulin sensitivity (8, 9). Therefore, more sophisticated secretory tests such as the glucose-potentiated arginine stimulation tests are used as measure of beta cell function capacity and a proxy for (functional) beta cell mass (10–12). It is also not possible to discern different beta cell secretory pathways using the MMTT. In-depth phenotyping of these cells using tests with intravenous stimuli can provide relevant pathophysiological information on the secretory capacity of the residual beta cell mass. It is also not known to what extent a MMTT, which is a more simple test to perform, can unmask residual beta cell function compared with more complex tests with intravenous stimuli.

Therefore, we studied the endogenous insulin secretory response to several relevant secretagogues in patients with longstanding type 1 diabetes and compared different tests for the unmasking of residual beta cell function.

## RESEARCH DESIGN AND METHODS

### Patients

Male patients with type 1 diabetes ≥18 yr, treated with insulin for at least 5 years, were recruited from the outpatient clinic of the Leiden University medical Center (LUMC). Male patients were chosen to exclude variable hormonal effects on different test days. Diagnosis of type 1 diabetes was made according to the ADA criteria (13). Type 1 diabetes was defined as a typical clinical course with rapid insulin treatment and positivity for at least one of the following autoantibodies: glutamic acid decarboxylase antibodies (anti-GAD), anti-tyrosine phosphatase-like protein (IA-2) autoantibodies, islet cell autoantibodies (ICA), and/or insulin autoantibodies (IAA) before starting insulin treatment. Exclusion criteria included HbA1c > 75 mmol/mol (>9%), BMI >30 kg/m^2^, known allergy against study medication, a history of cardiovascular disease, kidney disease (eGFR <60 mL/min/1.73 m^2^), liver disease, diseases of the central nervous system, or sickle cell disease. Furthermore, patients who used oral glucose lowering agents, medications that alter insulin sensitivity, systemic adrenergic agonists or antagonists, or potassium-sparing diuretics were excluded. There was no information on putative detectable C-peptide concentrations reflecting residual beta cell function at recruitment.

The study protocol was approved by the medical ethics committee of the LUMC and written informed consent was obtained from all subjects.

### Study Procedures

Subjects visited the clinical research unit in a fasting state twice within 4 wk from August 8, 2017 until April 4, 2018. Long acting insulin in patients using multiple daily injections or basal insulin rate in patients using continuous subcutaneous insulin infusion therapy were continued before and during the study visits. No short acting insulin boluses were allowed in the 4 h before the study visit.

During one visit, a three-phase hyperglycemic clamp was performed. During the other visit a MMTT was performed, including an intravenous arginine bolus at the end of the test. On both study visits subjects had to remain in a supine position during the test.

#### Three-phase hyperglycemic clamp.

When the subjects arrived at the clinical research unit, an intravenous cannula was inserted in both arms. One cannula intended for blood sampling was placed in retrograde fashion in a dorsal hand vein, and this hand was placed in a heated box (55°C) for arterialization of venous blood. The other intravenous cannula intended for the intravenous infusion was placed in an antecubital vein in the contralateral arm. Subsequently, glucose 20% (Baxter International, Deerfield, IL) and insulin aspart (NovoRapid, Novo Nordisk, Copenhagen, Denmark) infusion was started at a low rate (10 mL/h and 0.5 U/h, respectively).

Glucose was sampled frequently (every 5–10 min). Boluses of intravenous insulin were given if the glucose concentration was above 6 mmol/L, aiming for a plasma glucose concentration of 5 mmol/L. When the glucose concentration was below 6 mmol/L, the glucose infusion rate was adjusted to keep the glucose at ∼5 mmol/L.

**Figure 1.**
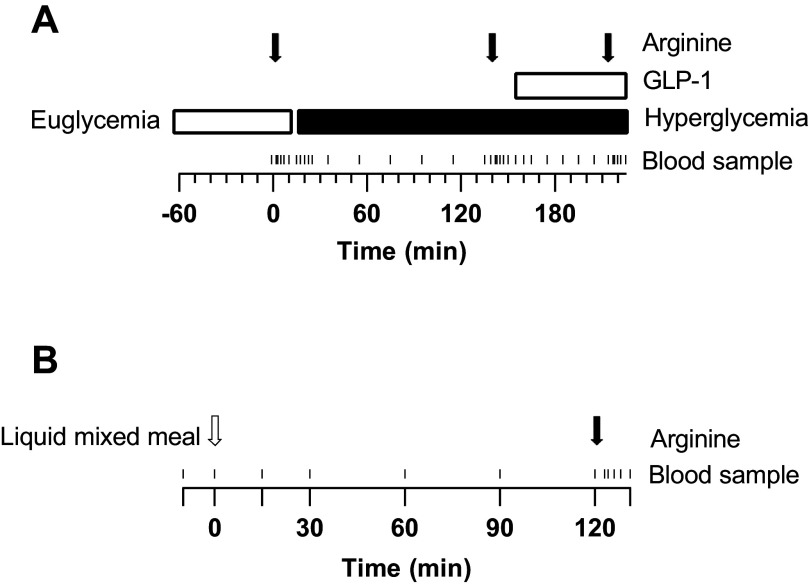
Graphical overview of the two study days. *A*: three-phase hyperglycemic clamp. Subjects first underwent a euglycemic clamp period (5 mmol/L) for at least 1 h after which an arginine bolus (5 g) was given at 0 min. Subsequently at 15 min a hyperglycemic phase was initiated (14 mmol/L), followed by another arginine bolus at 140 min. At 155 min, glucagon-like peptide 1 (GLP-1) infusion (4.5 mmol/kg bolus followed by 1.5 mmol/kg/min infusion) was started with another arginine bolus at 215 min. Blood samples were taken at 0, 15, 17.5, 20, 22.5, 25, 35, 55, 75, 95, 115, 135, 140, 155, 160, 165, 175, 185, 195, 205, and 215 min. In addition blood samples were taken at 2, 3, 5, 7, and 10 min after every arginine bolus. *B*: mixed meal tolerance test (MMTT). A liquid mixed meal (270 mL Nutridrink) was ingested at 0 min, followed by an arginine bolus at 120 min. Blood samples were taken at −10, 0, 15, 30, 60, 90, and 120 min. In addition, blood samples were taken 2, 3, 5, 7, and 10 min after the arginine bolus.

After at least 1 h of euglycemia, defined as glucose concentrations between 4 and 6 mmol/L, a 5 g arginine bolus was administered intravenously (at 0 min; Fig. 1). Fifteen minutes later, a body weight adapted glucose bolus was given to acutely raise the blood glucose to 14 mmol/L. This glucose concentration was maintained during the remainder of the clamp by adjusting the variable glucose infusion rate. At 140 min, another 5 g arginine bolus was administered and subsequently at 155 min a bolus of 4.5 pmol/kg glucagon-like peptide 1 (GLP-1) (Bachem, Weil am Rhein, Germany) was administered followed by continuous GLP-1 infusion rate of 1.5 pmol/kg/min for the remainder of the test. At 215 min a final 5 g arginine bolus was given.

Blood was regularly sampled throughout the test, with more frequent sampling after the arginine boluses and after the start of the glucose and GLP-1.

#### Mixed meal tolerance test.

At the beginning of this visit, a venous cannula was placed in the antecubital veins of each arm. Subsequently, subjects were instructed to ingest 270 mL of a liquid mixed meal (Nutridrink, Nutricia, Zoetermeer, The Netherlands, which consists of 49.7 g of carbohydrates, 15.9 g of protein and 15.7 g of fat). At 120 min, a 5 g bolus of arginine was administered intravenously. Frequent blood samples were taken (Fig. 1).

### Laboratory Assessments

All blood samples were put on ice directly after sampling, centrifuged within 1 h, and frozen at −80°C. The samples were thawed just before analysis. The samples were analyzed for C-peptide on Cobas e602 system (electrochemiluminescence immunoassay, Roche, Basel, Switzerland) with a detection limit of 0.003 nmol/L. The samples of the three-phase hyperglycemic clamp were also analyzed for glucagon (RIA, Merck Milipore, Burlington, MA; limit of quantification: 25 ng/L).

Total proinsulin (ELISA, Mercodia, Upsala, Sweden; detection limit: 3.55 pmol/L) was measured at all timepoints during the three-phase hyperglycemic clamp only in the participants in whom C-peptide was detectable at any time point. In the patients without detectable C-peptide, proinsulin was only measured at baseline and after 217 min, which is the timepoint, the highest proinsulin concentration was measured in the patients with detectable C-peptide.

During the three-phase hyperglycemic clamp, glucose was assessed at bedside with the Roche Accu-chek Inform II (Hoffman-La Roche, Basel, Switzerland) and by the hexokinase method on the COBAS 8000 (Hoffman-La Roche, Basel, Switzerland) during the MMTT.

### Statistics

The C-peptide response to a stimulus is presented as fold change compared with the prestimulatory C-peptide. During the three-phase hyperglycemic clamp, the secretory response to intravenous glucose was subdivided into a first phase response (maximal C-peptide*_t_*_= 15–25 min_ divided by C-peptide*_t_*_= 15 min_) and a second phase response (maximal C-peptide*_t_*_= 25–140 min_ divided by C-peptide*_t_*_= 15 min_). The secretory response to GLP-1 was calculated by maximal C-peptide*_t_*_= 155–215 min_ divided by C-peptide*_t_*_= 155_. During the MMTT, the secretory response to the meal was defined as maximal C-peptide*_t_*_= 0–120_ divided by C-peptide*_t_*_= 0 min_. The C-peptide responses to arginine were calculated as the maximal C-peptide concentration measured during the 10 min after the arginine bolus divided by the C-peptide concentration immediately before the bolus.

When poststimulatory C-peptide was detectable but prestimulatory C-peptide was not, the prestimulatory value was replaced by the lower limit of detection of the C-peptide assay (0.003 nmol/L) to be as conservative as possible. The C-peptide response was statistically tested by comparing the maximal C-peptide concentration after stimulation with the prestimulatory C-peptide concentration with a paired Student *t* test after logarithmic transformation.

The maximum C-peptide concentration after different stimuli during the three-phase hyperglycemic clamp and the mixed meal test was compared using the Student *t* test.

Glucagon responses are presented as fold change. The glucagon response to hyperglycemia was calculated by time weighted average glucagon*_t_*_= 15–135 min_ divided by glucagon*_t_*_= 0 min_, and the glucagon response to GLP-1 was calculated by time weighted average glucagon*_t_*_= 155–215 min_ divided by time weighted average glucagon*_t_*_= 15–135 min_. We chose for this approach using the time weighted average of the glucagon concentration to counter the known high variability of the glucagon assay (14). The glucagon responses to arginine were calculated identically to the C-peptide response to arginine. A considerable number of glucagon measurements (*n* = 171/540) were below the limit of quantification (25 ng/L) but we were able to extrapolate a lower estimate for most of them (*n* = 155). Besides the main analysis including the extrapolated lower estimates, we also performed a sensitivity analysis replacing all estimates lower than 25 ng/L with 25 ng/L.

A Student *T* test was used to compare the area under the glucagon curve and the fold changes of glucagon response to the different stimuli during the three-phase hyperglycemic clamp between the groups with detectable C-peptide at ≥1 time point and with undetectable C-peptide.

All analyses were performed using STATA version 14.1 (StataCorp LP, College Station, TX).

### Study Size

Using the data from Oram et al. (3), the logarithmically transformed SD of the C-peptide fold change was estimated (SD = 0.54). To reach 80% power to determine a C-peptide fold change of 2.0 with an alpha of 0.05, eight patients were needed. To account for undetectable C-peptide (27% in the aforementioned study), the inclusion of 15 patients was deemed sufficient.

## RESULTS

### Patient Characteristics

Fifteen male subjects were included with an average age of 34.9 ± 9.3 yr and a BMI of 24.9 ± 2.7 kg/m^2^. Type 1 diabetes had been diagnosed at 16.1 ± 9.9 yr of age and the diabetes duration was 18.7 ± 9.4 yr. The HbA1c was 58.6 ± 8.2 mmol/mol (7.5 ± 0.8%) and the subjects had a normal kidney function (eGFR 87.8 ± 9.8 mL/min/1.73 m^2^).

### Beta Cell Secretory Response to the Three-Phase Hyperglycemic Clamp

Data on glucose and insulin dosages during and before the test are presented in Supplemental Data and Supplemental Fig. S1, *A* and *B*.

**Figure 2.**
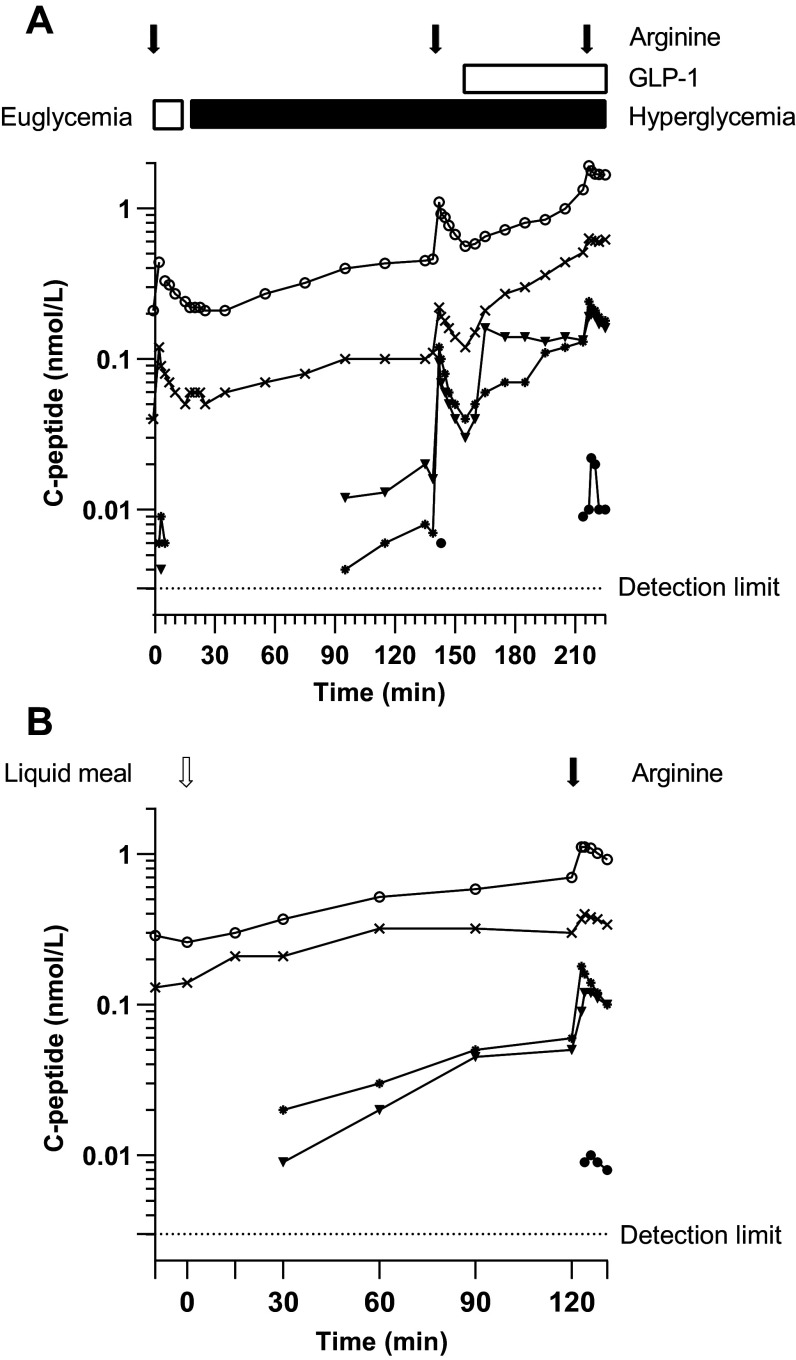
C-peptide concentrations during the clamp (*A*) and mixed meal tolerance test (MMTT; *B*). The curves represent the 5 of 15 individual participants in whom C-peptide was detectable at ≥1 time point. The filled circles (●) represent *subject 1*, the filled triangles (▾) *subject 4*, the open circles (○) *subject 6*, the asterisks (*) *subject 11*, and the crosses (×) *subject 14* in both subfigures. To also visualize low C-peptide concentrations, the curves are depicted on a logarithmic scale.

During the three-phase hyperglycemic clamp, baseline C-peptide was detectable in 2 of 15 subjects (*subject 6*: 0.21 nmol/L and *subject 14*: 0.04 nmol/L) (Fig. 2*A* and Supplemental Fig. S2*A*). In two other subjects (*subjects 4* and *11*), C-peptide could be detected after the first arginine bolus (at euglycemia) and after subsequent stimuli. In a fifth subject (*subject 1*), C-peptide was only detectable after the arginine bolus during the hyperglycemic phase and after GLP-1 stimulation.

When C-peptide was detectable in a subject (either at baseline or after stimulation), the hyperglycemia and GLP-1 infusion stimulated C-peptide, respectively, 2.9-fold [95% CI: 1.2–7.1, *P* = 0.03] and 3.5-fold [95% CI: 2.4–5.2, *P* < 0.001] (Fig. 2*A* and Supplemental Table S1). We did not observe a first-phase secretory response to hyperglycemia (1.05-fold [95% CI: 0.19–5.8, *P* = 0.78]). Arginine resulted in a C-peptide response during the three different clamp phases (ranging from a 1.6- to a 4.0-fold response). The maximal C-peptide reached during the test (after the arginine during hyperglycemia and GLP-1 infusion) was 22.3-fold [95% CI: 5.6–89.1, *P* = 0.003] higher than baseline C-peptide concentration or the limit of detection (when baseline C-peptide was undetectable).

Characteristics of patients with and without detectable C-peptide are presented in Supplemental Table S2.

### Beta Cell Secretory Response to the MMTT

Data on glucose and insulin dosages before the test are presented in Supplemental Data.

During the MMTT, glucose rose from 10.2 ± 3.1 mmol/L to a maximum of 20.1 ± 2.8 mmol/L (Supplemental Fig. S1*C*). Two subjects (*subjects 6* and *14*) had detectable fasting C-peptide concentrations during the MMTT (0.29 and 0.13 nmol/L, respectively). In two subjects, C-peptide became detectable during the MMTT (*subjects 4* and *11*) and one additional subject had detectable C-peptide only after the bolus of arginine (*subject 1*) (Fig. 2*B* and Supplemental Fig. S2*B*). The MMTT was able to elicit a 6.7-fold [95% CI: 1.1–42.4, *P* = 0.046] C-peptide response and arginine a subsequent 2.2-fold [95%: CI 1.3–3.6, *P* = 0.01] C-peptide response (at a glucose concentration of 19.3 ± 2.8 mmol/L) (Supplemental Table S1). Thus the MMTT plus intravenous arginine together showed a 10.0-fold [95% CI: 1.6–61.7, *P* = 0.03] response.

### Comparison of the Three-Phase Hyperglycemic Clamp and the MMTT

Two subjects (*subjects 6* and *14*) had detectable fasting C-peptide during both the three-phase hyperglycemic clamp and the MMTT. The two subjects (*subjects 4* and *11*) in which arginine or hyperglycemia was needed to have detectable C-peptide during the three-phase hyperglycemic clamp, also showed a C-peptide response during the MMTT. The subject (*subject 1*) that needed arginine or GLP-1 on top of hyperglycemia during the three-phase hyperglycemic clamp to unmask detectable C-peptide, showed detectable C-peptide only after the arginine bolus at the end of the MMTT. Ranking the peak C-peptide concentration for each visit resulted in the same order of subjects. When comparing the response between the two study visits, the C-peptide response to the MMTT was higher than the response to the hyperglycemic clamp (relative difference: 3.0 [95% CI: 1.04–8.7, *P* = 0.045]) but lower than the response to the hyperglycemic clamp combined with GLP-1 (relative difference: 0.5 [95% CI: 0.3–0.7, *P* = 0.01]). The response to arginine at the end of the MMTT showed the same pattern: higher than the response to arginine on top of hyperglycemia (relative difference: 1.4 [95% CI: 1.06–1.9, *P* = 0.03]) but lower than the response to arginine on top of hyperglycemia combined with GLP-1 (relative difference: 0.6 [95% CI: 0.5–0.7, *P* = 0.003]). There was a strong correlation between the C-peptide response to arginine on top of the MMTT and the response to arginine on top of the hyperglycemia (*r* = 0.99, *P* = 0.002).

### Alpha Cell Secretory Response to the Three-Phase Hyperglycemic Clamp

**Figure 3.**
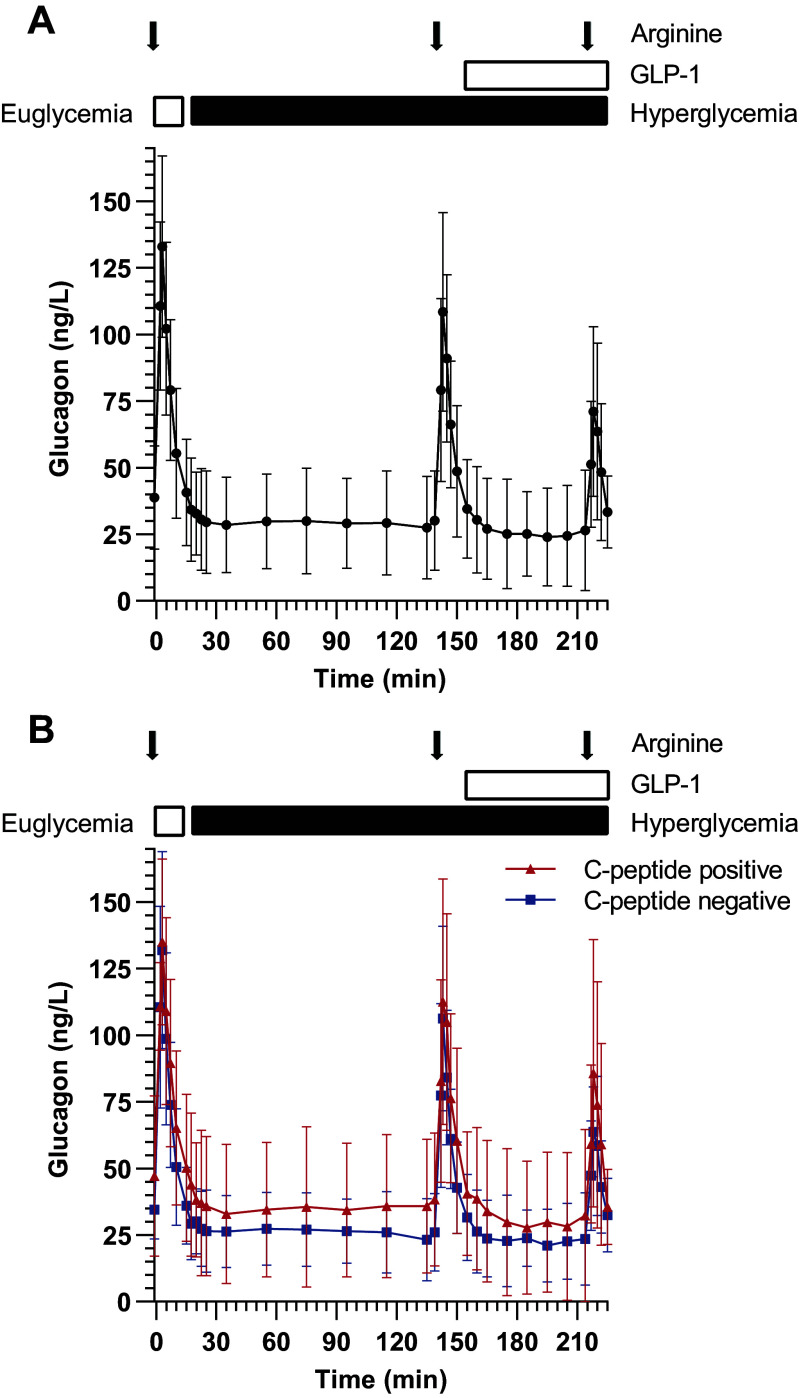
Glucagon during the three-phase hyperglycemic clamp. Both the average curve of the whole group (*A*) and the glucagon curve divided by C-peptide detectability (*B*) are depicted. The red triangles represent the C-peptide positive group and the blue squares the C-peptide negative group. The error bars represent standard deviation (SD).

Baseline glucagon was on average 38.8 ± 19.4 ng/L (Fig. 3*A*). During the three-phase hyperglycemic clamp, both glucose and GLP-1 infusion reduced plasma glucagon (respectively 0.72-fold [95% CI: 0.63–0.84, *P* < 0.001] and 0.85-fold [95% CI: 0.78–0.93, *P* = 0.002]) (Supplemental Table S3). Each arginine bolus resulted in a strong 3.3- to 4.1-fold increase in glucagon. The glucagon response to arginine (measured as the maximum glucagon concentration after the stimulus) was significantly lower during hyperglycemic phase (*P* < 0.001) compared with the euglycemic phase, and during hyperglycemia combined with GLP1 (*P* < 0.001) compared with only hyperglycemia.

The area under the glucagon curve during the three-phase hyperglycemic clamp was not different for subjects with detectable C-peptide and without detectable C-peptide (*P* = 0.47, Fig. 3*B*). There was also no difference between the two groups in the inhibition of glucagon secretion by hyperglycemia (C-peptide + group: 0.72 [95% CI: 0.60–0.86] fold, vs. C-peptide − group: 0.73, [95% CI: 0.58–0.91] fold, *P* = 0.92) or by GLP-1 (C-peptide positive group: 0.83 [95% CI: 0.66–1.05] fold, vs. C-peptide negative group: 0.86 [95% CI: 0.77–0.96] fold, *P* = 0.76).

Sensitivity analysis in which glucagon values <25 ng/mL were replaced with 25 ng/mL showed similar results (inhibition of glucagon secretion by hyperglycemia 0.85-fold [95% CI: 0.79–0.92] and by GLP-1 0.96-fold [95% CI: 0.92–0.997] in all subjects).

### Proinsulin Secretion

**Figure 4.**
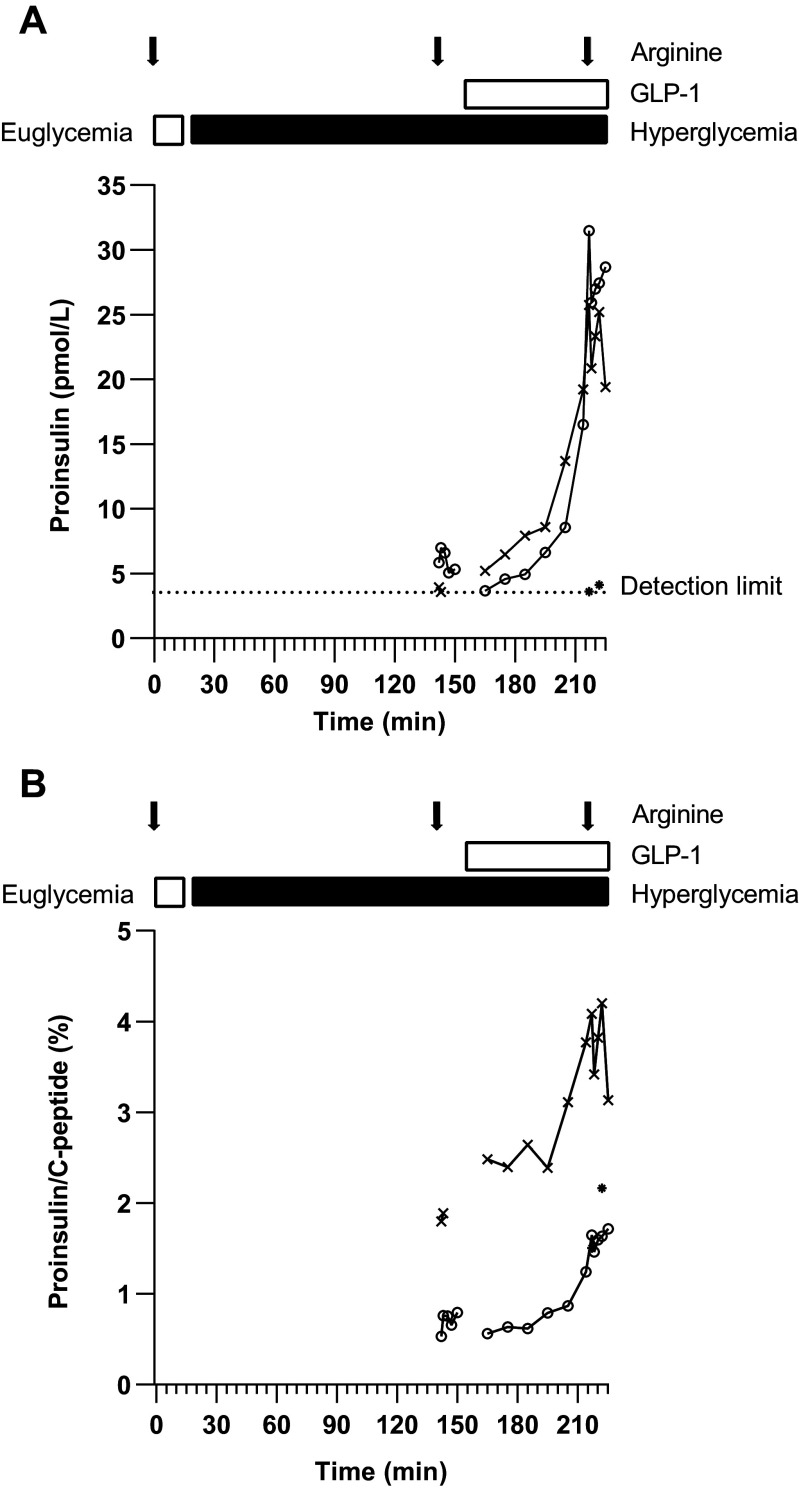
Proinsulin (*A*) and proinsulin to C-peptide ratio (*B*) during the three-phase hyperglycemic clamp. The curves represent the 3 of 15 individual participants in which proinsulin was detectable at ≥1 time point. The open circles (○) represent *subject 6*, the asterisks (*) *subject 11*, and the crosses (×) *subject 14* in both subfigures.

Proinsulin was only detectable in the three subjects with the highest stimulated C-peptide during the three-phase hyperglycemic clamp (Fig. 4). In those three subjects, proinsulin was only detectable after stimulation with hyperglycemia together with arginine and/or GLP-1. When proinsulin was detected, it mimicked the C-peptide response to the different stimuli. The proinsulin to C-peptide ratio was on average 2.0 ± 1.0% and the ratio increased after additional stimuli to a maximum of 2.7 ± 1.3%. In none of the C-peptide negative subjects during the two study visits, proinsulin could be detected at baseline or at maximal stimulation (hyperglycemia + GLP1 + arginine).

## DISCUSSION

Residual beta cells are often present in longstanding type 1 diabetes. To what extent different stimulatory and inhibitory pathways for insulin secretion in these residual beta cells are still functional and which tests can be used to unmask residual beta cell function is not clear. Therefore, we investigated the secretory response in patients with longstanding type 1 diabetes to different stimuli using a hyperglycemic clamp, GLP-1 infusion, and intravenous arginine boluses and compared this to a MMTT followed by an intravenous arginine bolus. We found that when C-peptide was detectable (fasting or after a specific stimulus), there was a C-peptide response to each subsequent stimulus. The relative responses to the stimuli, ranging from a 1.6- to 3.5-fold increase in C-peptide, were even comparable with the relative responses in a similar study performed with healthy subjects (15). The only exception was the first phase secretory response to hyperglycemia, which was never present in our subjects with type 1 diabetes.

Our results contrasts with a previous study in which a secretory response to a hyperglycemic clamp was only seen in the subset of subjects with MMTT stimulated C-peptide >0.4 nmol/L (12). This difference may be explained by the preceding phase of euglycemia (target glucose 5 mmol/L) for at least 1 h before the hyperglycemic phase, and the longer hyperglycemic phase (2 h instead of 45 min) in our study. Euglycemia can restore glucose responsiveness in isolated pancreatic islets from patients with type 1 diabetes (16).

The remaining beta cells could potentially contribute to glycemic control, since there is a response to different stimuli. However, whether there is some clinical benefit depends on the balance between the size of the functional residual beta cell mass and insulin requirement of the body. Clinical benefit can occur at even very low C-peptide levels of 5–30 pmol/L (7). It is unknown whether there is a clinical benefit of an even smaller functional beta cell mass and how this is related to insulin sensitivity. In addition, the evolution of residual beta cell function during the entire duration of type 1 diabetes could be related to the risk of complications. Future studies should address these issues. This knowledge can be used to assess the potential benefit of beta cell preservation or islet replacement strategies, in particular novel treatments using pluripotent stem cells (17).

To unmask remaining beta cell function using our sensitive C-peptide assay (lower limit of detection 0.003 nmol/L), a secretory stimulus was needed in three of five subjects. In two subjects, stimulation by either arginine, hyperglycemia, or the mixed meal was sufficient and in one subject a combination of stimuli was necessary (hyperglycemia + arginine, hyperglycemia + GLP-1 or a mixed meal + arginine). A major difference between our study and other studies performing beta cell stimulation tests in patients with type 1 diabetes (4, 5, 12) is that we did not only include patients with fasting or random detectable C-peptide. The proportion of participants with detectable C-peptide is in line with other studies when disease duration is taken into account (2, 4). The higher prevalence of patients with detectable C-peptide in some other studies could be related to other disease characteristics, indicated by lower autoantibody positivity (5) compared with our study with autoantibody positivity being related to a lower residual beta cell mass reserve (18).

Glucose-potentiated arginine-induced insulin secretion has been used as a marker for beta cell mass in hemi-pancreatectomized subjects and in islet transplant recipients (10, 19). In our study, the hyperglycemic clamp indeed caused an increased C-peptide response to arginine. But arginine in combination with a liquid meal yielded an even higher response and was highly correlated with arginine-induced C-peptide secretion during the hyperglycemic clamp. These are interesting observations from a clinical context point of view as the mixed meal stimulation plus intravenous arginine test is much less cumbersome than a hyperglycemic clamp for obtaining an indication of functional beta cell mass.

Abnormal proinsulin conversion to insulin has been implicated to be part of the pathogenesis of type 1 diabetes, with serum proinsulin being elevated relative to C-peptide before and shortly after diagnosis (20, 21). There has been a recent debate about the prevalence of proinsulin secretion in longstanding type 1 diabetes (22). We did not find detectable proinsulin in any of our subjects without detectable C-peptide and proinsulin was only detectable after a beta cell stimulus. Our data is therefore more in line with the prevalence of detectable proinsulin in the study of Steenkamp et al. (23) (16% in patient with undetectable postmeal C-peptide) compared with the T1D exchange residual C-peptide study group (89.9% in subjects without detectable C-peptide, and proinsulin not reacting to beta cell stimuli; 24). This could be due to the different proinsulin assay used (22).

Glucagon secretory dysfunction could also contribute to glycemic dysregulation in type 1 diabetes. During hypoglycemia, the glucagon response is impaired in patients with type 1 diabetes, whereas glucagon is inappropriately secreted after a meal (25, 26). In our study, glucagon secretion was inhibited by intravenous glucose, in both subjects with and without detectable C-peptide to a similar extent. But robust conclusions about the relevance of residual beta cell function for alpha cell function cannot be drawn from our study. Previously, Hare et al. (27) also observed that intravenous glucose inhibited glucagon release, whereas oral glucose resulted in the inappropriate increase of glucagon, pointing toward a potential role for gastrointestinal factors for the increase in glucagon release. We found that GLP-1 directly inhibited glucagon secretion, so GLP-1 sensitivity cannot explain the difference.

A limitation of the study is the small sample size due to the intensive protocols. However, because of the controlled conditions with frequent blood sampling, we were still able to distinguish statistically significant secretory patterns by most stimuli. Another limitation is the detection limits of the different assays for C-peptide, glucagon, and proinsulin even while using assays with a detection limit that are in line or lower compared with most other studies (2–5, 7, 12). As histological studies show that some insulin positive cells are present in most subjects with type 1 diabetes (1), one can expect detectable circulating C-peptide in the majority of subjects as long as the C-peptide assay is sensitive enough. And even very few residual beta cells are likely to be responsive to common secretory triggers if our findings can be extrapolated.

In conclusion, in patients with longstanding type 1 diabetes in whom residual beta cell function can be detected, several stimuli elicit a near-normal beta cell secretory response, except for the first phase insulin response to hyperglycemia. A relatively simple liquid mixed meal with a subsequent bolus of intravenous arginine would be a good test in clinical studies to detect the presence of minimal residual beta cell mass. What the minimal function of the residual beta cells should be in relation to insulin sensitivity to have a true clinical impact and thus is worth saving, still needs to be determined.

## DATA AVAILABILITY

The datasets generated during and/or analyzed during the current study are available from the corresponding author upon reasonable request.

## SUPPLEMENTAL DATA

10.17026/LS/07QNPT The Supplemental Data, Supplemental Figs. S1 and S2, and Supplemental Tables S1–S3: https://doi.org/10.17026/LS/07QNPT.

## GRANTS

This study has been partly funded by the Bontius Foundation.

## DISCLOSURES

A part of the results have been priorly published in abstract form for the Annual Dutch Diabetes Research Meeting 2018 and the annual Young Nederlands Vereniging voor Endocrinologie Conference (JNVE) 2018. No conflicts of interest, financial or otherwise, are declared by the authors.

## AUTHOR CONTRIBUTIONS

B.S.U., M.F.N., and E.J.P.d.K. conceived and designed research; B.S.U. performed experiments; B.S.U. analyzed data; B.S.U., M.F.N., and E.J.P.d.K. interpreted results of experiments; B.S.U. prepared figures; B.S.U. drafted manuscript; B.S.U., M.F.N., and E.J.P.d.K. edited and revised manuscript; B.S.U., M.F.N., and E.J.P.d.K. approved final version of manuscript.
